# Association between Regulated upon Activation, Normal T Cells Expressed and Secreted (RANTES) -28C/G Polymorphism and Susceptibility to HIV-1 Infection: A Meta-Analysis

**DOI:** 10.1371/journal.pone.0060683

**Published:** 2013-04-05

**Authors:** Zhenghua Gong, Jialin Tang, Tianxin Xiang, Lunli Zhang, Qinghua Liao, Wei Liu, Yalin Wang

**Affiliations:** 1 Department of Public Health, Center for Disease Control and Prevention in Jiangxi Province, Nanchang, China; 2 Departments of Infectious Diseases, the First Hospital Affiliated of Nanchang University, Nanchang, China; Imperial College London, United Kingdom

## Abstract

**Background:**

Many studies have investigated the distributions of RANTES genotypes between HIV-1 infected patients and uninfected individuals. However, no definite results have been put forward about whether the RANTES −28C/G polymorphism can affect HIV-1 susceptibility.

**Methods:**

We performed a meta-analysis of 12 studies including 7473 subjects for whom the RANTES −28C/G polymorphism was genotyped. Odds ratios (ORs) with 95% confidence intervals (CIs) were employed to assess the association of the polymorphism with HIV-1 susceptibility. By dividing the controls into healthy controls and HIV-1 exposed but seronegative (HESN) controls, we explored the both allelic and dominant genetic models.

**Results:**

By using the healthy controls, we found a marginally significant association between the −28C/G polymorphism and susceptibility to HIV-1 infection in the allelic model (OR = 0.82, 95%CI = 0.70–0.97). But sensitivity analysis suggested that the association was driven by one study. We further performed stratified analysis according to ethnicity. The −28G allele decreased susceptibility to HIV-1 infection in the allelic model among Asians (OR = 0.79, 95%CI = 0.66–0.94). By using the HESN controls, no association between the polymorphism −28C/G and the susceptibility to HIV-1 infection was revealed in either the allelic model (OR = 0.84, 95%CI = 0.60–1.17) or the dominant model (OR = 0.77, 95%CI = 0.54–1.10).

**Conclusions:**

Our findings suggested that the RANTES −28G allele might play a role in resistance to HIV-1 infection among Asians. Additional well-designed studies were required for the validation of this association.

## Introduction

Human immunodeficiency virus-1(HIV-1)/acquired immunodeficiency syndrome (AIDS) remains one of the world's most significant public health challenges. Up to now, nearly 34 million people live with HIV/AIDS all over the world, particularly in low and middle-income countries, and an estimated 2.7 million people were newly infected with the virus in 2010 (http://www.who.int/features/factfiles/hiv/en/). However, the susceptibility to infection after exposure and natural course of infection vary among individuals [1]. Nowadays, it has been generally accepted that genetic variants among individuals can regulate HIV-1 cell entry, immune responses and other factors that influence the susceptibility to HIV-1 infection, disease progression and curative effects [2]–[4].

Many studies have reported variants in the genes encoding HIV-1 coreceptors and their natural ligands, which have been shown to modify HIV-1 infection and disease progression [5]–[7]. Regulated upon activation, normal T cell expressed and secreted (RANTES, also named as CCL5), one of natural ligands for HIV-1 coreceptors, has been shown to play a role in immune responses to viral infections [8], [9]. While RANTES was originally considered a T cell-specific chemokine, it is now known to be expressed by a number of other cell types including epithelial cells and platelets and acts as a potent chemoattractant for many cell types such as monocytes, NK cells [10], memory T cells, eosinophils and DCs [11]. In combination with macrophage inhibitory proteins -1α and -1β, RANTES regulates protective immunity to HIV-1 infection by competing with HIV-1 envelope glycoprotein gp120 for binding to CC chemokine receptor 5 (CCR5). Sustained RANTES binding has the long term effects of reducing CCR5 surface levels [12].

The gene encoding RANTES which is located on chromosome 17q11.2-q12 includes three frequent polymorphisms: −403G/A and −28C/G in the promoter region, and IN1.1T/C in the first intron region [13]. The −28C/G polymorphism in the RANTES promoter region had been found to affect the transcription of the RANTES gene. In human cell lines, the −28G allele was shown to increase promoter activity of RANTES in comparison with the −28C allele, suggesting that the polymorphism can regulate RANTES expression in the human body and may delay HIV-1 disease progression [14]. Several molecular epidemiological studies have been conducted to examine the association between RANTES −28C/G polymorphism and susceptibility to HIV-1 infection. However the results remain controversial and inconclusive. Therefore a comprehensive analysis is critical.

To elucidate the role of the RANTES −28C/G polymorphism in HIV-1 infection, we performed a meta-analysis of all eligible related studies to obtain a decisive resolution, which may aid in understanding the level of risk of HIV-1 infection (Table S1).

## Materials and Methods

### Publication Search

We searched PubMed, Embase, and China National Knowledge Infrastructure for all articles on the association between RANTES polymorphisms and HIV-1 infection (last search update 1^st^ October 2012). The following key words were used: ‘HIV or AIDS or human immunodeficiency viruses’, ‘RANTES or CCL5’ and ‘polymorphism or variant’. The search was conducted with restriction on language in English and/or Chinese, also limited to human subjects. Reference lists of the identified articles were also examined and the literature retrieval was performed in duplicate by two independent reviewers (Gong and Tang).

### Inclusion and Exclusion Criteria

We reviewed abstracts of all citations and retrieved studies. The following criteria were used to include published studies: (a) case-control studies, regardless of sample size, that were conducted to evaluate the association between RANTES −28C/G polymorphism and the risk of HIV-1 infection, (b) studies that provided data on the distributions of −28C/G polymorphism in the case-control population, (c) studies that were published in English or Chinese languages. The major reasons for exclusion of studies were (a) duplication of previous studies, (b) reviews, (c) not human studies, (d) no cases or controls.

### Data Extraction

Two investigators (Gong and Tang) reviewed and extracted the information from all eligible publications independently according to the inclusion and exclusion criteria listed above. Disagreements were resolved by discussion between the two investigators. The following characteristics were collected from each study: first author, year of publication, country of studied population, ethnicity, number of HIV-1 patients, number of healthy controls, number of HIV-1 exposed but seronegative (HESN) controls, and the distributions of the RANTES −28C/G polymorphism in the cases and controls.

### Statistical Analysis

Crude odds ratios (ORs) with their 95% confidence intervals (CIs) were used to assess the strength of association between RANTES polymorphisms and susceptibility HIV-1 infections. Because the negligible frequency of genotype −28GG in the population, we didn't perform the homozygous model (GG vs. CC) and recessive model (GG vs. GC+CC) in this study. Both allelic model (G vs. C) and dominant model (GG+GC vs. CC) were conducted for the subgroup with both healthy controls and HIV-1 exposed but seronegative (HESN) controls. Analyses stratified by ethnicity were also performed. Heterogeneity assumption was assessed by χ^2^-based *Q*-test. If the *P* value for heterogeneity was >0.05, indicating an absence of heterogeneity between studies, we used the fixed-effect model (Mantel-Haenszel) to evaluate the summary ORs. In contrast, if the *P* value for heterogeneity was ≤0.05, indicating a high extent of heterogeneity between studies, we used the random-effect model (DerSimonian and Laird) to evaluate the summary ORs. The departure of frequencies of the RANTES polymorphism from expectation under Hardy-Weinberg equilibrium was assessed by the χ^2^ test in controls. Sensitivity analysis was performed by sequentially excluding individual studies to assess the stability of the results. Possible publication bias was tested by Begg's funnel plot and Egger's test.

All statistical tests were conducted with Stata software package (version 9.2, College Station, TX). A *P* value less than 0.05 was used to denote statistical significance.

## Results

### Study characteristics

**Figure 1:**
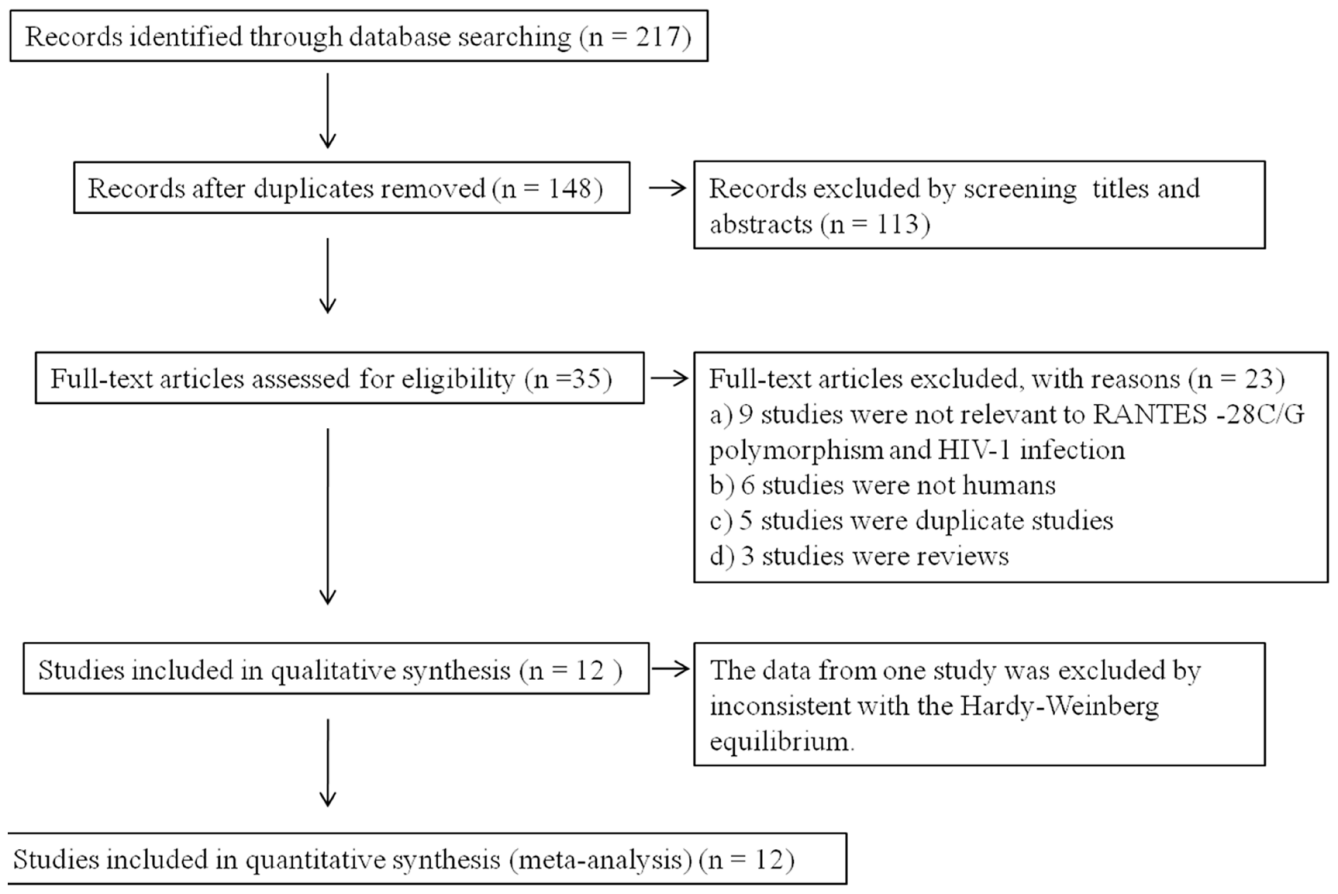
Flow diagram of study identification.

As shown in Figure 1, a total of 148 studies were identified after an initial search from the selected electronic databases. After screening the titles and abstracts, a total of 12 studies involved in three sub-populations were enrolled for meta-analysis [14]–[25]. Among them, there were seven studies for Asians, one study for Africans and six studies for Caucasians. All studies included healthy controls, while only five studies were conducted with HESN controls. The total number of samples involved in the 12 eligible studies was 7473, which included 3453 HIV-1 patients, 3682 healthy controls and 338 HESN controls. The genotype distributions in the controls among Africans in the study by Liu et al [15] significantly deviated from Hardy-Weinberg equilibrium; so the data among Africans in the study had been excluded. The characteristics of eventual included studies were listed in Table 1.

**Table 1:** Characteristics of the included studies in this meta-analysis.

First author [ref]^a^	Year	Country	Ethnicity	HIV-1 infected patients (GG/CG/CC)	Healthy controls (GG/CG/CC)	HESN^b^ controls (GG/CG/CC)
Liu [14]	1999	Japan	Asian	11/62/199	7/36/100	1/12/37
Liu [15]	1999	Japan, China, Thailand	Asian	0/12/56	9/60/200	
		France	Caucasian	0/3/65	0/2/28	
McDermott [16]	2000	NA^c^	Caucasian	0/18/331	0/10/141	0/2/77
Gonzalez [17]	2001	NA	African	0/6/397	0/1/459	
		NA	Caucasian	0/23/595	0/17/434	
Fernandez [18]	2003	Spain	Caucasian	0/28/412	0/6/94	
Zhao [19]	2004	China	Asian	1/48/200	25/224/779	
Liu [20]	2004	China	Asian	2/58/270	4/57/353	0/33/94
Ahlenstiel [21]	2005	Germany	Caucasian	0/13/186	0/6/103	
Vidal [22]	2006	Spain	Caucasian	0/8/147	0/8/90	
Suresh [23]	2006	India	Asian	0/0/50	0/2/73	0/1/34
Rathore [24]	2008	India	Asian	0/4/192	0/13/302	0/3/44
Jang [25]	2008	Korea	Asian	25/18/13	26/10/3	

a The ref was referred to the reference numbers in this study.

b HESN: HIV-1 exposed but seronegative.

c NA: Not available.

### Meta-analysis

After pooling the data from the 12 studies for meta-analysis, the results were calculated according to the allelic and dominant models.

**Figure 2:**
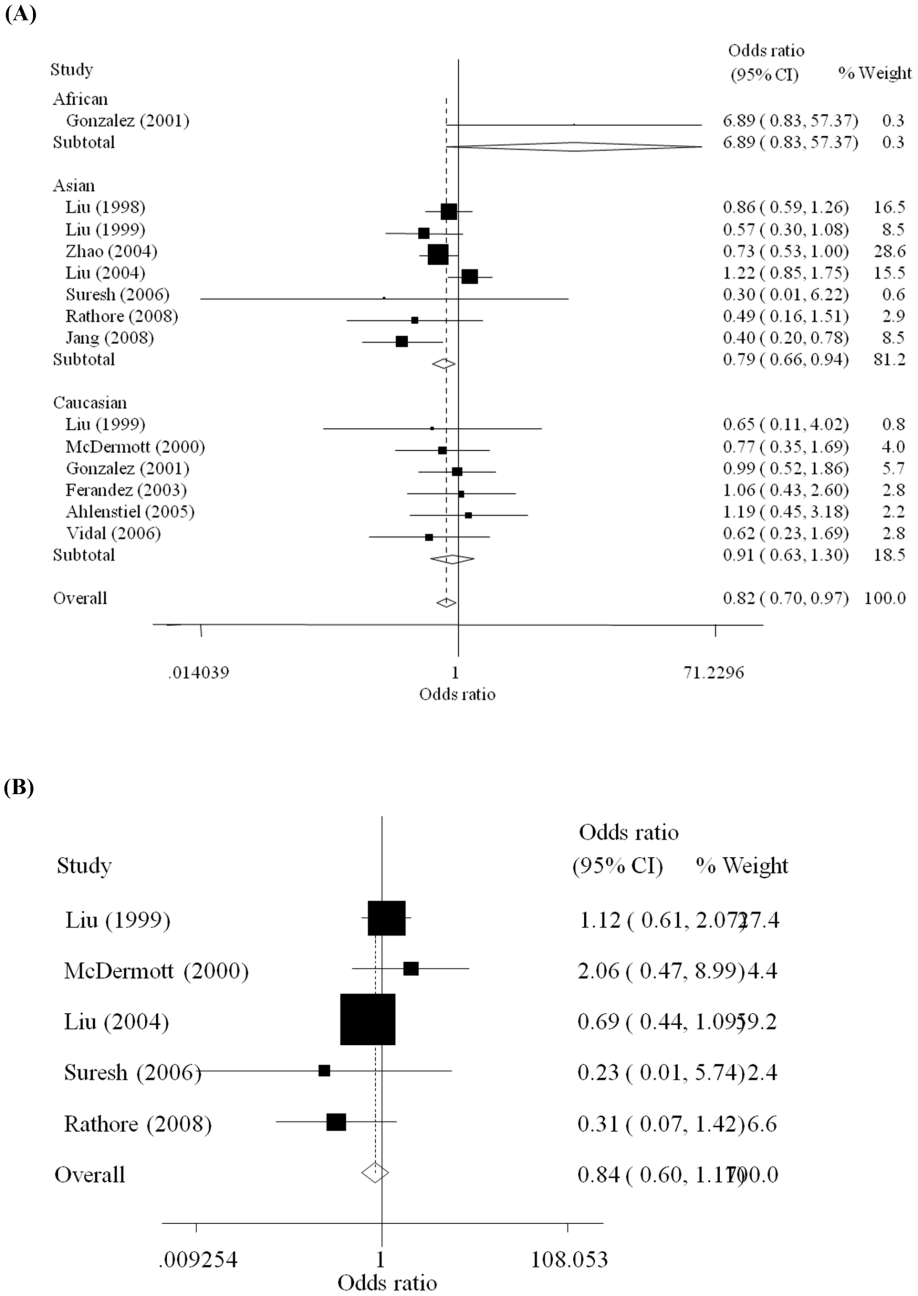
Forest plot of the association between RANTES −28C/G polymorphism and HIV-1 infection under the allelic model. (A)By using the healthy individuals as controls, the comparison which was stratified by ethnicity was carried out between the HIV-1 patients and healthy controls; (B) By using the HIV-1 exposed but seronegative (HESN) individuals as controls, the comparison was carried out between the HIV-1 patients and the HESN controls.

By using the healthy controls, we only found a marginally significant association between the −28C/G polymorphism and susceptibility to HIV-1 infection in the allelic model (G vs. C: OR = 0.82, 95%CI = 0.70–0.97, *P* = 0.175 for heterogeneity test). No association was found in the dominant model. We further performed stratified analysis according to ethnicity. The −28G allele decreased the susceptibility to HIV-1 infection in the allelic model among Asians (G vs. C: OR = 0.79, 95%CI = 0.66–0.94, *P* = 0.062 for heterogeneity test). The results were shown in Figure 2A.

By using the HESN controls, as shown in Figure 2B, no association between the polymorphism −28C/G and the susceptibility to HIV-1 infection was revealed in both allelic model (G vs. C: OR = 0.84, 95%CI = 0.60–1.17, *P* = 0.264 for heterogeneity test) and dominant model (GG+GC vs. CC: OR = 0.77, 95%CI = 0.54–1.10, *P* = 0.280 for heterogeneity test).

### Sensitivity Analysis

Sensitivity analysis was performed by deleting one study at one time to assess the stability of the pooled ORs. Sensitivity analysis was performed for various comparisons in the total population and all the subgroups. When one study among the Korean (Asian) population was deleted [25], the results in the overall population became statistically insignificant in the allelic model (OR = 0.86, 95% CI 0.73–1.02, *P* = 0.393 for heterogeneity test) by using the healthy controls. However, the results for the other models remained stable (Data were not shown).

### Publication bias

**Figure 3:**
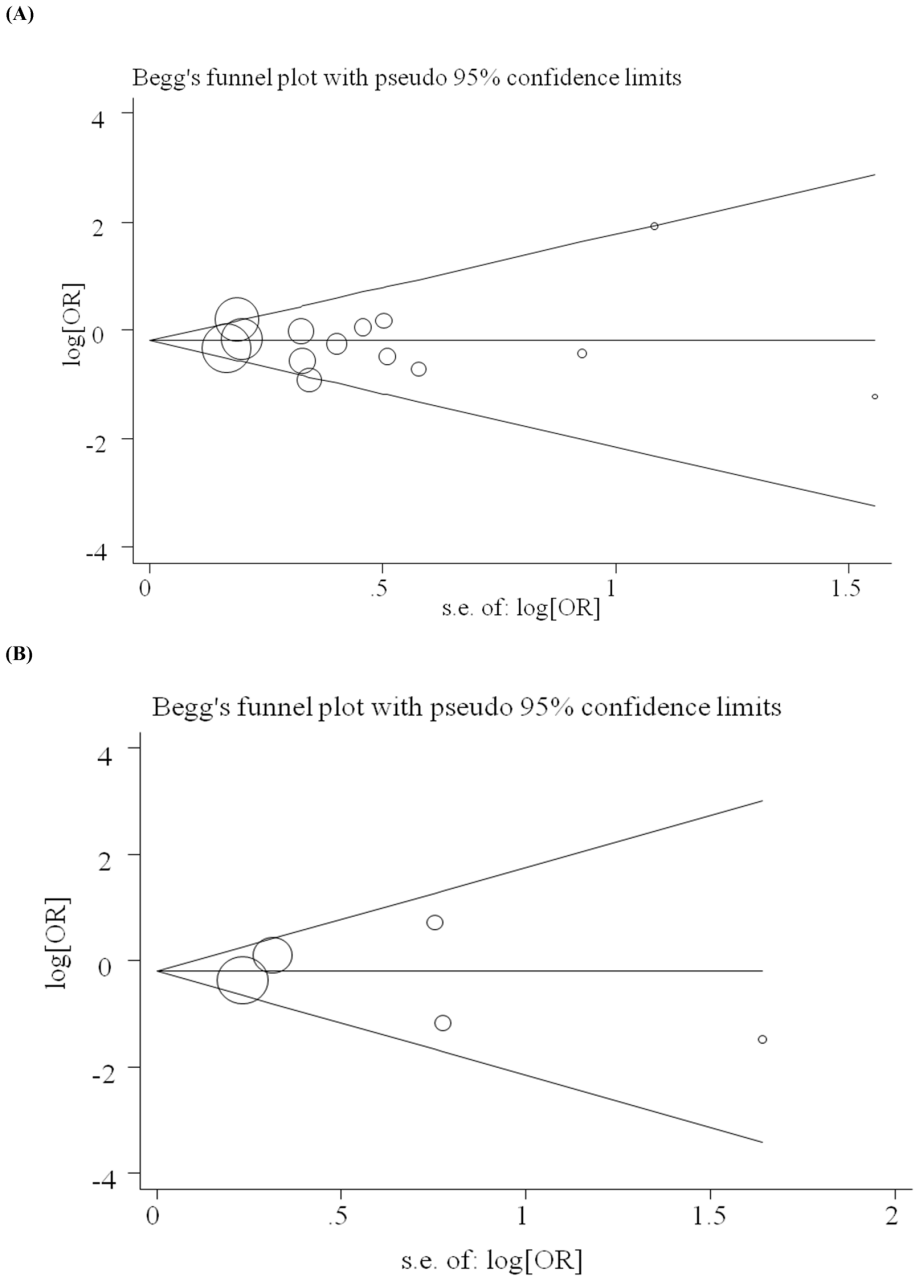
Funnel plots to detect publication bias in this meta-analysis. (A) Healthy controls considered; (B) HIV-1 exposed but seronegative controls considered. The horizontal line indicates the pooled log odds ratio (OR) and guidelines to assist in visualizing the funnel are pooled at 95% pseudo confidence limits for this estimate.

Visual inspection of funnel plot asymmetry was conducted. Also, Begg's rank correlation method and Egger's weighted regression method were used to statistically assess publication bias. There was no evidence of publication bias in the healthy controls group (Begg's test *P* = 1.00, Egger's test *P* = 0.82) (Figure 3A) or in the HESN controls group (Begg's test *P* = 0.81, Egger's test *P* = 0.69) (Figure 3B).

## Discussion

Many studies have investigated the associations between chemokine and chemokine-receptor gene polymorphisms and the susceptibility to HIV-1 infection. Recently a meta-analysis had demonstrated that the variable number tandem repeat (VNTR) allele polymorphism of Dendritic cell-specific intercellular adhesion molecule-3-grabbing nonintegrin related (DC-SIGNR) might have a role in resistance to HIV infection, particularly in Asian populations [26], while the other two studies had found that susceptibility of HIV-1 infection was not significantly affected by the polymorphisms CCR2-Val64Ile and CCR5-Δ32 [27], [28].

The RANTES-CCR5 pathway can influence immune responses in multiple ways during acute viral infections [29]–[31]. Humans with the CCR5-Δ32 genotype have slower progression with HIV infection [32] and therapeutic strategies targeting RANTES and CCR5 are being used for treatment against HIV infection [33]. A recent study had suggested that blocking the RANTES-CCR5 receptor pathway could alter the development and or quality of antiviral immune responses to chronic viral infection [34]. A previous study had indicated that the RANTES −28G allele has been shown to increase promoter activity and, thereby increasing the expression of RANTES protein and ultimately contributing to reduced CD4^+^ T lymphocyte depletion rates and slowed HIV-1 progression in affected individuals [14]. The frequency of −28G allele was low among Asians, but almost negligible in other ethnic groups [16], [17].The influence of the −28G allele on disease progression always depended on ethnicity, ranging from no effect in a multiethnic cohort [16], to slow progression in Japanese [14], [17], to rapid progression toward AIDS in Chinese [19]. Up to now, the association between the RANTES −28C/G polymorphism and the risk of HIV-1 infection had been illustrated in many reports, but the conclusions were controversial. Therefore we performed a meta-analysis which offered a powerful method to synthesize information of independent studies with similar intention.

The results involved in 12 eligible studies had suggested that the RANTES -28C/G polymorphism demonstrated a marginal association with the susceptibility to HIV-1 infection among Asians in the allelic model. The most probable explanation is that RANTES −28G could reduce the depletion rate of CD4^+^ T cells and increase RANTES expression in vivo.

Further in sensitivity analysis, we found the data from one study [25] significantly deviated from others. The allelic frequencies of RANTES −28G were 79.5% in the healthy controls and 60.3% in the HIV-1 infection patients among Koreans with an incredible high level in the study [25]. While in the other two included studies among East Asians, the allelic frequencies of RANTES −28G in the common population were 18.4% among Japanese [14] and 10.0% among Chinese [19] respectively. The samples in the study were similar with other included studies in this meta-analysis. And the methods applied had been reported previously in other article [23]. We finally included the study because it satisfied the inclusion criteria in this meta-analysis. As shown in Figure 2, the weight of the study on the Korean population was 8.5%. After the study [25] was deleted, just the association between the polymorphism and the susceptibility to HIV-1 infection was disappeared in the overall population as shown in the results. However, the results for the other models remained stable. Finally, the results still indicated that the −28G allele might decrease the susceptibility to HIV-1 infection among Asians in the allelic model.

It is now a consensus to define HESN individuals from several group of individuals who are at high risk of exposure which include : (1) commercial sex workers, (2) people with hemophilia, (3) discordant couples, (4) intravenous drug users, and (5) mother-to-child transmission [35]. By using the HESN controls, it looks like more powerful than using the random healthy individuals as controls. In further study, by using the HESN controls, we found no association between the RANTES −28C/G polymorphism and susceptibility to HIV-1 infection. The probable reasons are: (a) the sample size is small, only 5 eligible studies included which were only involved in 1197 HIV-1 cases and 481 controls, (b) the frequency of −28GG genotype was almost negligible among Africans and Caucasians, (c) the HIV-1 exposed but seronegative (HESN) individuals always included sub-populations such as commercial sex workers and so on. More studies are needed in the future among different ethnicities to explain the natural HIV-1 resistance in individuals exposed to HIV-1 who remain seronegative.

Although meta-analysis is a powerful statistical method, some limitations still exist here. First, the data of one included study significantly deviated from others which caused the unstable results; thus we should make conclusion more cautiously. Second, we only included the studies written in English and Chinese, and the related reports in other languages were not included, which might bias our conclusion in this study. Third, publication bias could not be excluded though the test showed negative results. The studies reporting significant associations between certain genotypes and reduced susceptibility to HIV infection would be more readily published while the studies with no significant associations would be more difficult to publish. Fourth, gene-gene and gene-environment interactions may influence host susceptibility to HIV-1 infection. In fact, many genes have been proven to influence HIV-1 infection risk, but we did not have enough data to eliminate these interfering factors. The prevalence of HIV-1 infection and progression is always related to the social economic status. Finally, further stratified analyses of patients and HESN individuals by infection exposure routes (sexual contact, intravenous drug use, etc.) could not be performed because the data detailing the infection route for the HIV-1 patients were lacking.

In conclusion, this meta-analysis involved in 12 case-control studies provided evidence that the RANTES −28G allele might play a role in resistance to HIV-1 infection among Asians. When the sensitivity analysis suggested that the results might be unstable sometimes, we should obtain this conclusion cautiously. Future studies in different ethnic populations and with clear infection routes should be performed to evaluate these associations.

## Supporting Information

Table S1 **PRISMA 2009 Checklist.**
(DOC)
