# Supplementary material for: Association between Regulated upon Activation, Normal T Cells Expressed and Secreted (RANTES) -28C/G Polymorphism and Susceptibility to HIV-1 Infection: A Meta-Analysis
Source: PLoS One. 2013 Apr 5;8(4):e60683. doi: 10.1371/journal.pone.0060683 (PMC3618220; doi:10.1371/journal.pone.0060683)
Supplement: Table S1 — PRISMA 2009 Checklist. (DOC) [file pone.0060683.s001.doc]

| **Section/topic** | **#** | **Checklist item** | **Reported on page #** |
| --- | --- | --- | --- |
| **TITLE** | | |  |
| Title | 1 | Association between regulated upon activation, normal T cells expressed and secreted (RANTES) -28C/G polymorphism and susceptibility to HIV-1 infection: a meta-analysis | 1 |
| **ABSTRACT** | | |  |
| Structured summary | 2 | **Background:** Many studies have investigated the distributions of RANTES genotypes between HIV-1 infected patients and uninfected individuals. However, no definite results have been put forward about whether the RANTES -28C/G polymorphism can affect HIV-1 susceptibility.  **Methods**: We performed a meta-analysis of 12 studies including 7473 subjects for whom the RANTES -28C/G polymorphism was genotyped. Odds ratios (ORs) with 95% confidence intervals (CIs) were employed to assess the association of the polymorphism with HIV-1 susceptibility. By dividing the controls into healthy controls and HIV-1 exposed but seronegative (HESN) controls, we explored the both allelic and dominant genetic models.  **Results**: By using the healthy controls, we found a marginally significant association between the -28C/G polymorphism and susceptibility to HIV-1 infection in the allelic model (OR = 0.82, 95%CI = 0.70-0.97). But sensitivity analysis suggested that the association was driven by one study. We further performed stratified analysis according to ethnicity. The -28G allele decreased susceptibility to HIV-1 infection in the allelic model among Asians (OR = 0.79, 95%CI = 0.66-0.94). By using the HESN controls, no association between the polymorphism -28C/G and the susceptibility to HIV-1 infection was revealed in either the allelic model (OR = 0.84, 95%CI = 0.60-1.17) or the dominant model (OR = 0.77, 95%CI = 0.54-1.10).  **Conclusions:** Our findings suggested that the RANTES -28G allele might play a role in resistance to HIV-1 infection among Asians. Additional well-designed studies were required for the validation of this association. | 2 |
| **INTRODUCTION** | | |  |
| Rationale | 3 | Several molecular epidemiological studies have been conducted to examine the association between RANTES -28C/G polymorphism and susceptibility to HIV-1 infection. However the results remain controversial and inconclusive. | 3-4 |
| Objectives | 4 | To elucidate the role of the RANTES -28C/G polymorphism in HIV-1 infection, we performed a meta-analysis of all eligible related studies to obtain a decisive resolution, which may aid in understanding the level of risk of HIV-1 infection. | 4 |
| **METHODS** | | |  |
| Protocol and registration | 5 | None |  |
| Eligibility criteria | 6 | The following criteria were used to include published studies: (a) case-control studies, regardless of sample size, were conducted to evaluate the association between RANTES -28C/G polymorphism and risk of HIV-1 infection, (b) the studies provided data on the distributions of -28C/G polymorphism in the case-control population | 5 |
| Information sources | 7 | We searched PubMed, Embase, and China National Knowledge Infrastructure for all articles on the association between RANTES polymorphisms and HIV-1 infection (last search update 1^st^ October 2012). | 5 |
| Search | 8 | The following key words were used: ‘HIV or AIDS or human immunodeficiency viruses’, ‘RANTES or CCL5’ and ‘polymorphism or variant’. The search was conducted with restriction on language in English and/or Chinese, also limited to human subjects. | 5 |
| Study selection | 9 | Two investigators (Gong and Tang) reviewed and extracted the information from all eligible publications independently according to the inclusion and exclusion criteria listed above. Disagreements were resolved by discussion between the two investigators. | 5 |
| Data collection process | 10 | Two investigators (Gong and Tang) reviewed and extracted the information from all eligible publications independently according to the inclusion and exclusion criteria listed above. Disagreements were resolved by discussion between the two investigators. | 5 |
| Data items | 11 | first author, year of publication, country of studied population, ethnicity, number of HIV-1 patients, number of healthy controls, number of HIV-1 exposed but seronegative (HESN) controls, and the distributions of the RANTES -28C/G polymorphism in the cases and controls. | 5 |
| Risk of bias in individual studies | 12 | Possible publication bias was tested by Begg’s funnel plot and Egger’s test. | 6 |
| Summary measures | 13 | Crude odds ratios (ORs) with their 95% confidence intervals (CIs) were used to assess the strength of association between the gene RANTES polymorphisms and HIV-1 infections. | 6 |
| Synthesis of results | 14 | Both allelic model (G vs. C) and dominant model (GG+GC vs. CC) were conducted. Heterogeneity assumption was assessed by χ^2^-based *Q*-test. | 6 |

Page 1 of 2

| **Section/topic** | **#** | **Checklist item** | **Reported on page #** |
| --- | --- | --- | --- |
| Risk of bias across studies | 15 | Publication bias, selective reporting within studies. | 6 |
| Additional analyses | 16 | Sensitivity analysis was performed by sequentially excluding individual studies to assess the stability of the results. Also stratified analyses were performed by ethnicity. | 6 |
| **RESULTS** | | |  |
| Study selection | 17 | The details were performed by a flow diagram as shown in Figure 1. | 7 |
| Study characteristics | 18 | The details were performed in section ‘Study characteristics’. | 7 |
| Risk of bias within studies | 19 | Begg’s rank correlation method and Egger’s weighted regression method were used to statistically assess publication bias. There was no evidence of publication bias in the healthy controls group (Begg’s test *P* = 1.00, Egger’s test *P* = 0.82) (Figure 3A) or in the HESN controls group (Begg’s test *P* = 0.81, Egger’s test *P* = 0.69) (Figure 3B). | 8 |
| Results of individual studies | 20 | The details were performed in section ‘Meta-analysis’. The major results were also performed by forest plot Figure 2. | 7 |
| Synthesis of results | 21 | The details were performed in section ‘Meta-analysis’. | 7-8 |
| Risk of bias across studies | 22 | No significant bias was observed across studies. | 8 |
| Additional analysis | 23 | Sensitivity analysis was performed for various comparisons in the total population and all the subgroups. One study might be the major reason for between heterogeneity. The details were presented in section ‘sensitivity analyses’. | 8 |
| **DISCUSSION** | | |  |
| Summary of evidence | 24 | The results involved in 12 eligible studies had proved that the RANTES -28C/G polymorphism demonstrated a marginal association with the susceptibility to HIV-1 infection among Asians in the allelic model. | 9-11 |
| Limitations | 25 | First, the data of one included study significantly deviated from others which caused the unstable results; thus we should make conclusion more cautiously. Second, we only included the studies written in English and Chinese, and the related reports in other languages were not included, which might bias our conclusion in this study. Third, publication bias could not be excluded though the test showed negative results. The studies reporting significant associations between certain genotypes and reduced susceptibility to HIV infection would be more readily published while the studies with no significant associations would be more difficult to publish. Fourth, gene-gene and gene-environment interactions may influence host susceptibility to HIV-1 infection. In fact, many genes have been proven to influence HIV-1 infection risk, but we did not have enough data to eliminate these interfering factors. The prevalence of HIV-1 infection and progression is always related to the social economic status. Finally, further stratified analyses of patients and HESN individuals by infection exposure routes (sexual contact, intravenous drug use, etc.) could not be performed because the data detailing the infection route for the HIV-1 patients were lacking. | 11 |
| Conclusions | 26 | In conclusion, this meta-analysis involved in 12 case-control studies provided evidence that the RANTES -28G allele might play a role in resistance to HIV-1 infection among Asians. When the sensitivity analysis suggested that the results might be unstable sometimes, we should obtain this conclusion cautiously. Future studies in different ethnic populations and with clear infection routes should be performed to evaluate these associations. | 11-12 |
| **FUNDING** | | |  |
| Funding | 27 | This work was supported by National Natural Science Foundation of China (No. 81060127).The funders had no role in study design, data collection and analysis, decision to publish, or preparation of the manuscript. |  |

*From:*  Moher D, Liberati A, Tetzlaff J, Altman DG, The PRISMA Group (2009). Preferred Reporting Items for Systematic Reviews and Meta-Analyses: The PRISMA Statement. PLoS Med 6(6): e1000097. doi:10.1371/journal.pmed1000097

For more information, visit: **www.prisma-statement.org**.

Page 2 of 2
